# Another Texas Medical College

**Published:** 1894-09

**Authors:** 


ANOTHER TEXAS MEDICAL COLLEGE.--THE JOURNAL has been favored with the announcement of the first session of the *Medical Department of the University of Fort Worth*, and it is a great surprise to us, inasmuch as we were not aware that such a movement was on foot. The medical department of the Fort Worth University seems to have sprung into the arena full fledged —like Minerva from the brain of Jove, and fully equipped for the contest for supremacy and students. It makes a most creditable showing, and it is claimed by the management that there are ample and first-class facilities for clinical study and for anatomical purposes. The large railroad hospital and the St. Joseph's Infirmary and some other institutions are under the management of the staff, and their college building is said to be well adapted for the purpose of teaching medicine. If *ability* on the part of the faculty were the only or chief requisite to success, success is already assured, for we can say with truth and without hesitation, that most of the gentlemen comprising the faculty are men of ripe experience and recognized ability in their several departments. The professors have all been appointed from the local profession of Fort Worth, and the faculty is a strong one, and all of them will make able teachers beyond doubt. Fort Worth ought to be a good location for a medical college, as it is central, and is a great railroad town, not less than ten roads centering, crossing or terminating there.

THE JOURNAL commends the pluck and enterprise of the incorporators, and wishes them abundant success. Of the wisdom, necessity or expediency of the undertaking we are not prepared to speak.

For a catalogue giving all information, address Professor B. Saunders, M. D., Dean, Fort Worth, Texas.

